# Opioidverordnungen bei Versicherten ohne Krebserkrankung in Deutschland – Daten der BARMER

**DOI:** 10.1007/s00482-024-00852-8

**Published:** 2024-12-05

**Authors:** Veronika Lappe, Daniel Grandt, Ursula Marschall, Frank Petzke, Winfried Häuser, Ingrid Schubert

**Affiliations:** 1 PMV Forschungsgruppe an der Medizinischen Fakultät und Universitätsklinikum Köln, Universität zu Köln, Köln, Deutschland; 2 Klinikum Saarbrücken, Innere Medizin 1, Saarbrücken, Deutschland; 3 Abteilungsleitung Medizin und Versorgungsforschung BARMER, Kompetenzzentrum Medizin, Wuppertal, Deutschland; 4 Klinik für Anästhesiologie, Schmerzmedizin, Universitätsmedizin Göttingen, Göttingen, Deutschland; 5 Klinikum rechts der Isar, Klinik für Psychosomatische Medizin und Psychotherapie, Technische Universität München, München, Deutschland; 6 Medizinisches Versorgungszentrum für Schmerzmedizin und seelische Gesundheit, Saarbrücken St. Johann, Saarbrücken, Deutschland

**Keywords:** Nichttumorbedingte Schmerzen, Schmerztherapie, Opioid-Langzeittherapie, Komedikation, Versorgungsforschung, Non-cancer pain, Pain therapy, Opioid long-term therapy, Comedikation, Health services research

## Abstract

**Hintergrund:**

Der Stellenwert der Opioide bei nichttumorbedingten Schmerzen wird kontrovers diskutiert. Aus Deutschland fehlen aktuelle Daten zur Opioidverordnung bei nichttumorbedingtem Schmerz.

**Ziel der Arbeit:**

Daten zur Prävalenz von kurz- und langfristigen Opioidverordnungen, verschriebenen Wirkstoffen, Komedikation, verschreibenden Fachgruppen und demografischen und klinischen Charakteristika der Patienten.

**Material und Methoden:**

Retrospektive Analyse von Abrechnungsdaten erwachsener BARMER-Versicherter ohne Hinweis auf einen bösartigen Tumor für das Jahr 2021 (*n* = 6.771.075) sowie Versicherter mit Neubeginn einer Opioidtherapie in 2019 (*n* = 142.598).

**Ergebnisse:**

5,7 % der Versicherten ohne Krebsdiagnose erhielten in 2021 mindestens eine Opioidverordnung, 1,9 % eine Langzeittherapie. Tilidin und Tramadol waren die am häufigsten verordneten Opioide in Kurz- und Langzeittherapie. Frauen erhielten häufiger Opioide als Männer. Die Verordnungshäufigkeit stieg mit dem Alter deutlich an. In 2021 erhielten 22,5 % der Versicherten mit Langzeitopioidtherapie eine Komedikation mit Pregabalin und/oder Gabapentin, 37,5 % mit einem Antidepressivum und 58,1 % mit Metamizol und/oder nichtsteroidale Antirheumatika (NSAR). Erstverordnungen erfolgten zu 59,5 % durch Hausärzte. Im ersten Therapiejahr waren bei Personen mit Langzeitopioidtherapie im Mittel 2,1 Praxen an der Schmerzmittelverordnung beteiligt, 13 verschiedene chronische Krankheiten wurden dokumentiert.

**Diskussion:**

Die Opioidtherapie nichttumorbedingter Schmerzen findet überwiegend im hausärztlichen Bereich bei älteren, multimorbiden Patienten statt. Die Indikationsstellung erfordert eine gemeinsame Entscheidungsfindung mit Patientinnen und Patienten und gegebenenfalls ihren Angehörigen sowie die Überprüfung möglicher Arzneimittelinteraktionen.

**Graphic abstract:**

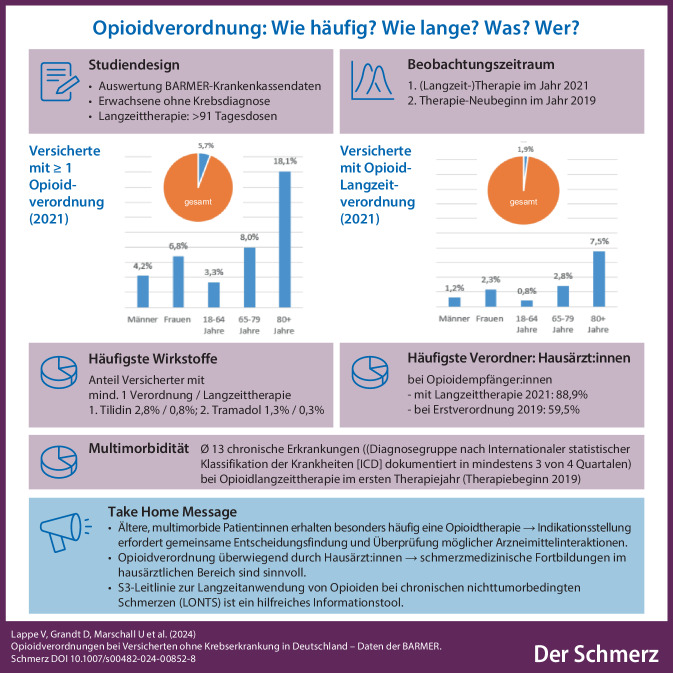

**Zusatzmaterial online:**

Die Online-Version dieses Beitrags (10.1007/s00482-024-00852-8) enthält weitere Tabellen.

## Abstract (en)

**Background:**

The importance of opioids in the treatment of non-cancer pain is under debate. No current data are available from Germany on the prevalence of opioid treatment for non-cancer pain.

**Aim of the study:**

Data on the prevalence of short- and long-term opioid prescriptions for patients without cancer, prescribed agents, co-medication, specialty of prescribing physicians, demographic and clinical characteristics of patients.

**Materials and methods:**

Retrospective analysis of billing data of adult BARMER-insured persons without evidence of cancer (*N* = 6,771,075) in 2021 and for patients initiating opioid therapy in 2019 (*n* = 142,598).

**Results:**

In total, 5.7% of the insured persons without a cancer diagnosis received at least one prescription for an opioid in 2021, while 1.9% received long-term therapy. Tilidine and tramadol were the most frequently prescribed opioids in short- and long-term therapy. Women received opioids more frequently than men. The frequency of prescriptions significantly increased with age. In 2021, 22.5% of insured persons with long-term opioid therapy received a co-medication with pregabalin and/or gabapentin, 37.5% with an antidepressant and 58.1% with metamizole and/or NSAIDs. A total of 59.5% of first prescriptions were issued by general practitioners. In the first year of therapy, an average of 2.1 practices were involved in prescribing analgetics for people on long-term opioid therapy and 13 different chronic diseases were documented.

**Discussion:**

Opioid therapy for non-cancer-related pain is predominantly carried out by general practitioners in older and multi-morbid patients. The indication for or against opioid therapy requires shared decision-making with patients and, if necessary, their relatives, as well as a review of possible drug interactions.

## Einleitung

Die Opioidepidemie beziehungsweise Opioidkrise, d. h. der parallele Anstieg von Opioidverordnungen, notfallmäßigen Krankenhausaufnahmen wegen Überdosierungen und Intoxikationen, missbräuchlicher Verwendung sowie Todesfällen erschütterte Nordamerika zwischen 1999 und 2010 [23]. Die aktuell vierte Welle der Opioidkrise in den USA sind überwiegend Todesfälle durch illegal hergestelltes Fentanyl [5]. Mehrere Analysen haben gezeigt, dass es in Deutschland keine Opioidkrise gibt [15, 26]. Auch die deutsche Bundesregierung stellte am 15.04.2019 auf eine Anfrage der FDP [9] und am 07.01.2020 auf eine Anfrage der Linken hin fest [8], dass es in Deutschland keine Hinweise für eine Opioidepidemie wie in den USA gibt. Die Bundesregierung führte als Gründe an, dass im Gegensatz zu den USA in Deutschland Schmerzfreiheit nicht zu einem Standard bei der Honorierung von Krankenhausleistungen gemacht wurde und es keine Verordnungsanreize für Ärzte gab. Weiterhin wurde auf die deutlich restriktiveren Verordnungsregelungen im Betäubungsmittelrecht in Deutschland verwiesen. Aus Sicht der Deutschen Schmerzgesellschaft hat auch die von ihr koordinierte S3-Leitlinie zur Langzeitanwendung von Opioiden bei chronischen nichttumorbedingten Schmerzen (LONTS), welche erstmals 2009 erschien [25] und 2015 [13] sowie 2020 [14] aktualisiert wurde, zu einem verantwortungsvolleren Umgang mit Opioiden beigetragen [6].

Während die grundsätzliche Bedeutung von Opioiden in der Behandlung perioperativer Schmerzen [7], in der Tumorschmerztherapie und anderen palliativen Indikationen unbestritten ist, wird ihr Einsatz zur Behandlung akuter [19, 24] und chronischer nichttumorbedingter Schmerzen [4] weiterhin kritisch diskutiert, vor allem wegen der potenziellen Risiken von Opioiden, z. B. der Kombination mit anderen zentralwirksamen Analgetika [22], Überdosierung bei Leber- und Niereninsuffizienz [20] oder des erhöhten Risikos missbräuchlicher Verwendung bei der Verschreibung durch mehrere Ärzte [21]. Die letzten uns bekannten deutschlandweiten Daten zu Langzeitopioidverordnungen für nichttumorbedingte Schmerzen in Deutschland stammen aus dem Jahr 2014 [21].

Zur Beantwortung der oben genannten potenziellen Probleme der Arzneimittelsicherheit werden Daten des Arzneimittelreports der BARMER 2023 [11] ergänzt um weitere Auswertungen berichtet:

- Anzahl der Versicherten ohne Hinweis auf eine Krebserkrankung mit mindestens einer Opioidverordnung und mit Langzeitverordnung
- Am häufigsten verordnete Opioide, demografische Charakteristika (Alter, Geschlecht, Begleiterkrankungen) und verordnende Facharztgruppen
- Kombinationstherapie von Opioiden mit Antikonvulsiva, Antidepressiva und Nichtopioidanalgetika bei Versicherten mit Langzeitopioidtherapie
- Bei Neubeginn der Opioidtherapie: initiierende Facharztgruppen sowie Anzahl im ersten Therapiejahr verordnender und behandelnder Ärzte und Ärztinnen und medizinische Charakteristika (Multimorbidität) der Versicherten mit Langzeitverordnungen

## Methoden

### Studienpopulationen

Für die Studie werden die folgenden Studienpopulationen betrachtet:

#### Studienpopulation 1 „Versicherte im Jahr 2021“.

Mit dieser Studienpopulation wurde die Verordnung von Opioiden bei erwachsenen BARMER-Versicherten im Einjahreszeitraum 2021 untersucht. Es wurden in 2021 durchgängig versicherte Personen ab 18 Jahren ohne Krebsdiagnose (ICD-Codes C00 bis C97 „Bösartige Neubildungen“) im Beobachtungsjahr eingeschlossen. Bei in 2021 Verstorbenen wurde das Jahr vor dem Versterben ausgewertet bei durchgängiger Versicherung in diesem Zeitraum. Da Pflichtversicherte einen nachgehenden Leistungsanspruch von einem Monat nach Versicherungsende haben, wenn dann nicht zum Beispiel die Familienversicherung greift, wurden Versicherte mit einer Versicherungslücke von bis zu 31 Tagen als durchgängig versichert eingestuft.

#### Studienpopulation 1a „Versicherte im Jahr 2021 durchgängig versichert ab 2017“.

Für die Teilpopulation der Studienpopulation 1, die von 2017 bis 2021 durchgängig versichert war, wurde die Opioidtherapie im Fünfjahreszeitraum analysiert. Bei Versicherten, die in 2021 verstarben, wurde der Fünfjahreszeitraum vor dem Versterben betrachtet.

#### Studienpopulation 2 „Versicherte mit Langzeitopioidtherapie im Jahr 2021“.

Diese Studienpopulation ist die Untergruppe der Studienpopulation 1, die in 2021 eine Langzeitopioidtherapie hatte entsprechend der unten aufgeführten Definition.

#### Studienpopulation 2a „Versicherte mit Langzeitopioidtherapie im Jahr 2021 durchgängig versichert ab 2017“.

Analog zur Studienpopulation 1a wurde die Opioidtherapie im Fünfjahreszeitraum bei der Teilpopulation der Studienpopulation 2, die von 2017 bis 2021 durchgängig versichert war, betrachtet.

#### Studienpopulation 3 „Versicherte mit inzidenter Opioidtherapie im Jahr 2019“.

Mit dieser Studienpopulation wurden die im Jahr 2019 neu begonnene Schmerzmedikation mit Opioiden und der Verordnungsverlauf im ersten Therapiejahr bei erwachsenen BARMER-Versicherten ohne Krebsdiagnose analysiert, die von 2017 bis 2021 durchgängig versichert waren. Von einer erstmaligen Opioidschmerztherapie wurde ausgegangen, wenn in den zwei Jahren zuvor kein Opioid verordnet wurde.

#### Studienpopulation 3a „Versicherte mit inzidenter Opioidtherapie im Jahr 2019 mit Langzeittherapie im ersten Therapiejahr“.

Es handelt sich um die Untergruppe von Studienpopulation 3, die eine Langzeitopioidtherapie im ersten Therapiejahr hatte.

## Medikation und Definition Langzeitopioidtherapie

Es wurde die im ambulanten Sektor verordnete Schmerzmittelmedikation betrachtet, die nach dem Apothekenabgabedatum zeitlich zugeordnet wurde. Im stationären Sektor verabreichte Medikamente konnten mit den Daten nicht abgebildet werden. Ebenso gilt das für Rezepte, die ausgestellt, aber nicht eingelöst wurden.

Bei den Opioiden (Anatomisch-therapeutisch-chemische Klassifikation: ATC-Code N02A) wurde (Levo‑)Methadon (ATC-Codes N02AC06/-52) nicht berücksichtigt, da die Wirkstoffe in der Regel zur Substitution bei der Suchttherapie verwendet werden und nur in Ausnahmefällen zur Schmerztherapie. Opioide der ATC-Code-Gruppe N07BC „Mittel zur Behandlung der Opiatabhängigkeit“ wurden ebenfalls nicht berücksichtigt.

Laut LONTS-Leitlinie wird eine Langzeitanwendung von opioidhaltigen Analgetika aus klinischer Sicht bei einer Therapiedauer von über drei Monaten angenommen [6]: Entsprechend wurde eine Behandlung mit mehr als 91 Tagesdosen (entsprechend „defined daily dose“ [DDD]) im Beobachtungsjahr 2021 sowie einem Abstand zwischen Einlösung der ersten und letzten Verordnung von mindestens 91 Tagen als Langzeittherapie definiert. Bei der Verordnung verschiedener Opioidwirkstoffe wird die Anzahl der jeweiligen DDD addiert. Damit nicht eine kurzzeitige Verordnung in hoher Dosis als Langzeitgebrauch definiert wird, wird zusätzlich zum Kriterium mehr als 91 DDD der Mindestabstand von 91 Tagen verlangt. Die DDD-Angaben sind der Quelle „Amtliche Fassung des ATC-Index mit DDD-Angaben für Deutschland im Jahre 2023“ entnommen [1].

Für Versicherte mit einer Langzeitopioidtherapie in 2021 wurde eine überlappende Therapie von mindestens 14 sowie mindestens 91 Tagen anhand der verordneten Tagesdosen („defined daily dose“ [DDD]) ab dem Apothekenabgabedatum für folgende Wirkstoffe beziehungsweise Wirkstoffgruppen betrachtet:

- Antikonvulsiva: Pregabalin (ATC-Code N03AX16), Gabapentin (N03AX12)
- Antidepressiva: ATC-Code N06A
- Nichtopioide: Hier wurden die Gruppen „nichtsteroidale Antiphlogistika und Antirheumatika“ (NSAR; ATC-Code M01A) und Metamizol (N02BB02) analysiert.

Manche ATC-Codes verschlüsseln Kombinationen mehrerer Wirkstoffe. Alle namentlich genannten Wirkstoffe dieser Kombinations-ATC-Codes wurden mit den ATC-Codes der Einzelwirkstoffe verschlüsselt und bei der Auswertung berücksichtigt. Pflanzliche und homöopathische Wirkstoffe wurden nicht in die Auswertung einbezogen. Es wurde nicht nach verschiedenen Darreichungsformen (Tabletten, Tropfen etc.) unterschieden.

## Verordnende und behandelnde Arztpraxen sowie Fachgebiete

Die Anzahl der an der Verordnung und Behandlung beteiligten Arztpraxen wurde anhand der pseudonymisierten Betriebsstättennummern (BSNR) erfasst. Laborärzte (Fachgruppenschlüssel [FG] 48) wurden nicht zu den behandelnden Arztpraxen gerechnet, da in der Regel nur die Abrechnung der Laborleistungen über die Praxen stattfindet und kein direkter Arztkontakt erfolgt, was ebenso für die Fachgebiete Mikrobiologie (FG 49) und Pathologie (FG 55, 56) gilt. Die Fachgebiete der verordnenden Ärztinnen und Ärzte wurden über die pseudonymisierte lebenslange Arztnummer (LANR) ermittelt, die für jede Ärztin beziehungsweise jeden Arzt eindeutig ist. Sie enthält an der achten und neunten Stelle den separat in den Daten verfügbaren Fachgruppenschlüssel, dem das Fachgebiet entnommen werden kann. Zusammengefasst wurden FG 01, 02, 03 zu Hausärztin/Hausarzt, FG 06–09, 11–14, 50, 52 zu Chirurgie, FG 44, 51, 53 zu Neurologie, FG 23, 32 zu Innere Medizin, FG 47, 58–61, 68, 69 zu Psychiatrie/Psychotherapie und FG 19, 20 zu Hals-Nasen-Ohren-Heilkunde.

## Multimorbidität von Versicherten mit Langzeitopioidtherapie im ersten Therapiejahr

Es wurde untersucht, wie viele unterschiedliche Diagnosegruppen im ersten Therapiejahr in mindestens drei von vier Quartalen dokumentiert waren, um chronische beziehungsweise über einen längeren Zeitraum behandelte Erkrankungen abzubilden. Dabei wurden die ab dem Quartal des Beginns der Therapie im Jahr 2019 dokumentierten Diagnosegruppen analysiert. Um Erkrankungen statt Einzeldiagnosen abzubilden, wurden dabei die 241 verschiedenen Krankheitsgruppen des ICD-Codes verwendet, bei denen dreistellige ICD-Codes zu sinnvollen Gruppen zusammengefasst sind, zum Beispiel ICD E10–E14 zu „Diabetes mellitus“ [2].

## Ergebnisse

### Opioidtherapie bei Versicherten ohne Krebsdiagnose im Jahr 2021

Im Jahr 2021 erfüllten 7.585.462 erwachsene BARMER-Versicherte das Einschlusskriterium der durchgängigen Versicherung. Bei 89,3 % (6.771.075 Versicherten) ergab sich aufgrund der codierten Diagnosen kein Hinweis auf eine Krebserkrankung im Beobachtungsjahr. Diese BARMER-Versicherten ohne Krebsdiagnose sind Grundlage der Auswertungen zur Opioidtherapie in 2021 (Studienpopulation 1) und erhielten zu 5,7 % (388.095 Versicherte) in diesem Jahr mindestens eine Verordnung eines Opioids. Geht man von allen 511.838 in 2021 durchgängig versicherten Erwachsenen aus, die in 2021 mindestens eine Opioidverordnung hatten, war bei 75,8 % keine Krebsdiagnose kodiert.

#### Opioidverordnungen nach Therapiezeitraum und in Abhängigkeit von Alter und Geschlecht

**Abb. 1:**
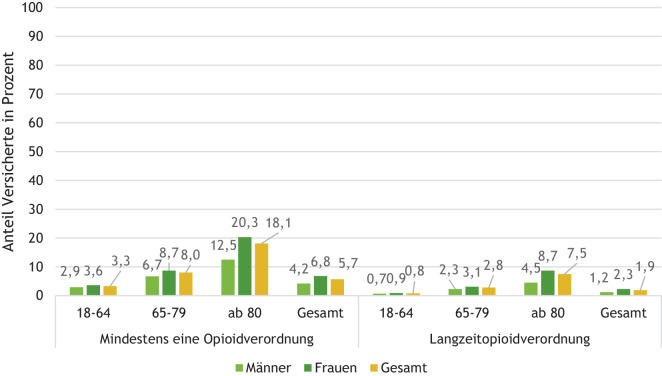
Anteil Versicherter ab 18 Jahre mit mindestens einer Opioidverordnung beziehungsweise Langzeitopioidtherapie im Jahr 2021. Quelle: BARMER-Daten 2020–2021; Studienpopulation 1: BARMER-Versicherte 2021 ohne Krebsdiagnose ab 18 Jahre: gesamt *n* = 6.771.075, Männer *n* = 2.857.793, Frauen = 3.913.282

Frauen erhielten häufiger Opioidverordnungen als Männer. Das galt in allen Altersgruppen und sowohl insgesamt als auch bei Langzeittherapie (siehe Abb. 1 und 2). Die Häufigkeit der Verschreibungen nahm mit dem Alter deutlich zu.

**Abb. 2:**
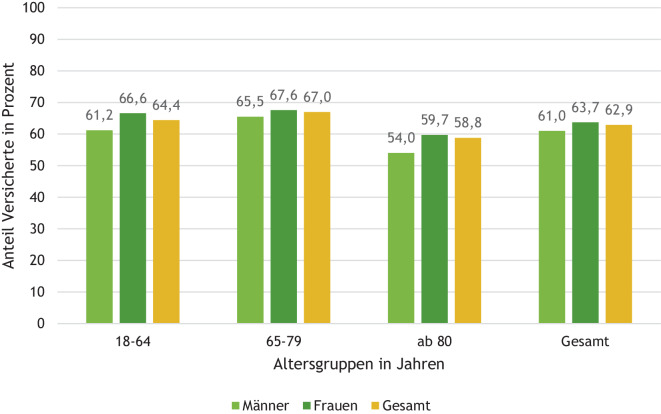
Versicherte mit Langzeitopioidtherapie in 2021 – Anteil mit Verordnung im gesamten Fünfjahreszeitraum 2017–2021. Quelle: BARMER-Daten 2017–2021; Studienpopulation 2a: BARMER-Versicherte 2021 ohne Krebsdiagnose ab 18 Jahre mit Langzeittherapie von Opioiden in 2021, durchgängig versichert 2017 bis 2021, Altersgruppe 18–64 *n* = 33.753, 65–79 *n* = 33.829, 80 + *n* = 44.778, gesamt *n* = 112.360

Blickt man vom Jahr 2021 vier Jahre zurück und ermittelt bei Versicherten mit Langzeitopioidtherapie in 2021 (Studienpopulation 2a) den Anteil der Versicherten, die auch schon in jedem der vier Jahre davor Opioide verordnet bekamen, zeigt sich, dass 62,9 % dieser Versicherten auch in den Jahren 2017 bis 2020 bereits Opioidverordnungen erhalten hatten. Bezogen auf alle erwachsenen BARMER-Versicherten, die im Fünfjahreszeitraum durchgängig versichert waren, erhielten damit 1,2 % eine Dauer- oder zumindest Intervalltherapie mit Opioiden. Die Zunahme mit dem Alter ist hier geringer ausgeprägt als bei der 1‑Jahres-Betrachtung und geht ab dem Alter von 80 Jahren zurück.

#### Häufigste im Jahr 2021 verordnete Opioide

Tilidin (2,8 %) und Tramadol (1,3 %) wurden im Jahr 2021 am häufigsten verordnet (siehe Tab. 1).

**Tab. 1:** Häufigste bei Erwachsenen verordnete Opioidschmerzmittel im Jahr 2021

ATC-Code	Wirkstoff*	Anteil Versicherte mit mindestens einer Verordnung in 2021 in Prozent		
		Gesamt	Männer	Frauen
N02AX01	Tilidin	2,8	2,2	3,3
N02AX02	Tramadol	1,3	1,0	1,5
N02AA05	Oxycodon	0,6	0,4	0,8
N02AB03	Fentanyl	0,4	0,2	0,6
N02AA59	Codein	0,4	0,3	0,4
N02AA03	Hydromorphon	0,3	0,2	0,4
N02AX06	Tapentadol	0,3	0,2	0,4
N02AA01	Morphin	0,3	0,2	0,3
N02AE01	Buprenorphin	0,2	0,1	0,3

*Wirkstoffe, die mindestens ein Promille der Versicherten in 2021 verordnet bekamen. Wirkstoffe von Kombinations-ATC-Codes werden einzeln bei den entsprechenden ATC-Codes berücksichtigt. Quelle: BARMER-Daten 2020–2021; Studienpopulation 1: BARMER-Versicherte 2021 ohne Krebsdiagnose ab 18 Jahre, gesamt *n* = 6.771.075, Männer *n* = 2.857.793, Frauen *n* = 3.913.282

Auch in der Langzeitanwendung waren Tilidin (0,8 % der Versicherten) und Tramadol (0,3 % der Versicherten) die am häufigsten verordneten Opioide. Der Anteil der in Langzeittherapie Behandelten war insbesondere bei Fentanyl (46,7 %) und Hydromorphon (44,4 %) hoch, während nur bei 20,6 % der Versicherten mit einer Tramadolverordnung diese auch als Langzeittherapie fortgeführt wurde (siehe Tab. 2).

**Tab. 2:** Häufigste bei Erwachsenen verordnete Opioidschmerzmittel in Langzeittherapie im Jahr 2021

ATC-Code	Wirkstoff*	Anteil Versicherte mit Langzeittherapie des Wirkstoffs in 2021 in Prozent					
		An allen Versicherten			An Versicherten mit mindestens einmaliger Verordnung dieses Wirkstoffs		
		Gesamt	Männer	Frauen	Gesamt	Männer	Frauen
N02AX01	Tilidin	0,8	0,5	1,0	27,3	24,4	28,7
N02AX02	Tramadol	0,3	0,2	0,3	20,6	19,3	21,2
N02AA05	Oxycodon	0,2	0,2	0,3	34,1	35,2	33,6
N02AB03	Fentanyl	0,2	0,1	0,3	46,7	43,0	47,6
N02AA03	Hydromorphon	0,1	0,1	0,2	44,4	43,8	44,6
N02AX06	Tapentadol	0,1	0,1	0,1	37,3	37,2	37,3
N02AE01	Buprenorphin	0,1	0,0	0,1	28,9	31,7	28,1

*Wirkstoffe, die mindestens ein Promille der Versicherten in 2021 verordnet bekamen. Wirkstoffe von Kombinations-ATC-Codes werden einzeln bei den entsprechenden ATC-Codes berücksichtigt. Quelle: BARMER-Daten 2020–2021; Studienpopulation 1: BARMER-Versicherte 2021 ohne Krebsdiagnose ab 18 Jahre, gesamt *n* = 6.771.075, Männer *n* = 2.857.793, Frauen *n* = 3.913.282

Bei den über den Fünfjahreszeitraum 2017 bis 2021 mit Opioiden therapierten Versicherten mit Langzeitverordnung in 2021 (Studienpopulation 1a) waren ebenfalls Tilidin und Tramadol die häufigsten eingesetzten Wirkstoffe (siehe Tabelle E1 im Online-Zusatzmaterial).

#### Verordnende Fachgruppen bei Langzeittherapie in 2021

Am häufigsten erhielten Versicherte mit Langzeittherapie im Jahr 2021 Verordnungen von Hausärzten (siehe Tab. 3), insbesondere in den höheren Altersgruppen.

**Tab. 3:** Anteil der Versicherten mit Langzeitopioidtherapie in 2021 nach Fachgebiet der verordnenden Ärztinnen und Ärzte

Fachgebiet	Anteil Versicherter mit Langzeitopioidtherapie nach Fachgebiet der verordnenden Ärzte					
	Alle Altersgruppen			Beide Geschlechter nach Altersgruppen in Jahren		
	Gesamt	Männer	Frauen	18–64	65–79	80+
	Prozent	Prozent	Prozent	Prozent	Prozent	Prozent
Hausärztin/Hausarzt	88,9	88,1	89,2	82,8	87,2	94,4
Anästhesiologie	10,7	10,9	10,6	17,3	11,6	5,4
Orthopädie	8,8	8,8	8,8	10,4	10,3	6,6
Chirurgie	4,1	4,6	3,9	5,9	4,4	2,5
Unbekannt	3,7	3,7	3,7	3,7	3,4	3,9
Neurologie	3,0	3,4	2,9	4,5	3,4	1,8
Innere Medizin	1,6	1,5	1,6	1,0	1,6	1,9
Physikalische/rehabilitative Medizin	1,2	1,2	1,3	1,8	1,4	0,8
Rheumatologie	1,0	0,8	1,1	1,5	1,3	0,5
Nephrologie	0,8	1,1	0,7	0,7	1,0	0,7
Psychiatrie/Psychotherapie	0,5	0,6	0,5	1,0	0,4	0,2
Zahnheilkunde	0,4	0,4	0,4	0,7	0,4	0,2
Sonstige Fachgebiete	2,8	2,9	2,7	3,4	2,5	2,5

Mehrfachnennungen möglich. Quelle: BARMER-Daten 2020–2021; Studienpopulation 2: BARMER-Versicherte 2021 ohne Krebsdiagnose ab 18 Jahre mit Langzeitopioidtherapie in 2021, gesamt *n* = 126.188, Männer *n* = 34.834, Frauen *n* = 91.354

#### Komedikation mit Antikonvulsiva, Antidepressiva und Nichtopioidanalgetika bei Versicherten mit Langzeitopioidtherapie im Jahr 2021

In 2021 erhielten 22,5 % der Versicherten mit Langzeitopioidtherapie eine Komedikation mit Pregabalin oder Gabapentin, 37,5 % mit einem Antidepressivum und 58,1 % mit Metamizol und/oder NSAR. Pregabalin/Gabapentin sowie Antidepressiva erhielten 3,6 % für mindestens 14 Tage parallel zu den Opioiden. Von den Versicherten mit Langzeitopioidtherapie erhielten 16,2 % in 2021 zu verschiedenen Zeiten oder gleichzeitig eine Komedikation sowohl mit Antidepressiva als auch mit NSAR/Metamizol. 7,3 % erhielten als Komedikation alle drei untersuchten Wirkstoffgruppen (siehe Tab. 4).

**Tab. 4:** Komedikation mit Antikonvulsiva, Antidepressiva beziehungsweise Nichtopioidanalgetika bei Versicherten mit Langzeitopioidtherapie in 2021

Geschlecht		Anteil Versicherte mit Langzeitopioidtherapie in 2021 und Komedikation* mit …								
		dem Wirkstoff/der Wirkstoffgruppe					nur den aufgeführten Wirkstoffgruppen in 2021			
		(Weitere Wirkstoff[-gruppen] möglich)					Zu verschiedenen Zeiten oder gleichzeitig			
	Altersgruppe	Pregabalin/Gabapentin	Antidepressiva	NSAR	Metamizol	NSAR/Metamizol	Preg./Gabp. Antidepress.	Preg./Gabp. NSAR/Metamizol	Antidepressiva NSAR/Metamizol	Preg./Gabp. Antidepressiva NSAR/Metamizol
Männer	18–64	15,8	27,8	19,1	7,6	25,2	4,7	2,7	5,5	2,8
	65–79	14,7	20,7	13,0	9,6	21,6	3,3	2,7	4,1	1,6
	80+	9,8	14,8	6,7	13,4	19,1	1,4	2,2	3,2	0,8
	Gesamt	14,0	22,2	14,0	9,7	22,5	3,4	2,5	4,4	1,9
Frauen	18–64	16,3	37,2	22,6	8,0	28,6	5,4	2,6	8,7	3,5
	65–79	11,6	26,2	14,3	9,7	22,7	3,0	2,0	5,5	1,8
	80+	6,6	20,1	6,7	15,3	20,9	1,1	1,5	5,5	0,8
	Gesamt	10,4	26,0	12,7	11,9	23,3	2,7	1,9	6,3	1,7
Gesamt	Gesamt	11,4	24,9	13,0	11,3	23,0	2,9	2,1	5,8	1,8

*Parallele Verordnung über mindestens 14 Tage. Quelle: BARMER-Daten 2020–2021; Studienpopulation 2: BARMER-Versicherte 2021 ohne Krebsdiagnose ab 18 Jahre mit Langzeitopioidtherapie in 2021, gesamt *n* = 126.188, Männer *n* = 34.834, Frauen *n* = 91.354

Die Komedikation fand häufig über einen längeren Zeitraum von mindestens 91 Tagen statt. Von den Versicherten mit Langzeitopioidtherapie erhielten 11,4 % eine Komedikation über mindestens 91 Tage mit Pregabalin und/oder Gabapentin, 24,9 % mit Antidepressiva und 23,0 % mit NSAR/Metamizol (siehe Tabelle E2 im Online-Zusatzmaterial).

### Opioidtherapie im ersten Therapiejahr bei Versicherten ohne Krebsdiagnose mit Therapiebeginn im Jahr 2019

Bei den Versicherten mit neu beginnender Opioidtherapie in 2019 wurden ebenfalls nur Versicherte ohne Krebsdiagnose betrachtet. Es waren insgesamt 142.598 Personen, 49.759 Männer und 92.839 Frauen (Studienpopulation 3). Von diesen hatten 12.690 Personen, 3768 Männer und 8922 Frauen, eine Langzeitopioidtherapie im ersten Therapiejahr (Studienpopulation 3a).

#### Die Opioidtherapie initiierende Fachgruppen

Die Opioiderstverordnungen wurden zu 59,5 % von Hausärzten und -ärztinnen ausgestellt, bei Patientinnen und Patienten ab 80 Jahren sogar zu 73,7 % (siehe Tabelle E3 im Online-Zusatzmaterial).

#### Anzahl der an der Verordnung und Behandlung bei Langzeittherapie im ersten Therapiejahr beteiligten Arztpraxen

**Abb. 3:**
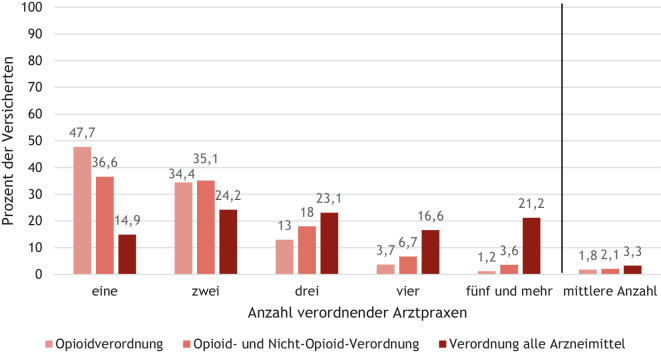
Anzahl an der Schmerzmittel- und Gesamtverordnung beteiligter Arztpraxen bei inzidenten Opioidpatienten mit Langzeittherapie im ersten Therapiejahr. Quelle: BARMER-Daten 2017–2021; Studienpopulation 3a: BARMER-Versicherte ohne Krebsdiagnose ab 18 Jahren mit Langzeitopioidtherapie im ersten Jahr ab Inzidenz in 2019, durchgängig versichert 2017 bis 2021, gesamt *n* = 12.690, Männer *n* = 3768, Frauen *n* = 8922

Nur 36,6 % der Patientinnen und Patienten mit Langzeittherapie im ersten Therapiejahr erhielten Verordnungen von opioid- und nichtopioidhaltigen Schmerzmitteln innerhalb eines Jahres nach Beginn einer langfristigen Opioidtherapie von nur einer Ärztin oder einem Arzt. Betrachtet man ausschließlich die Verordnung von Opioiden waren es 47,7 %. Im Mittel waren 2,1 Praxen an der Opioid- beziehungsweise Schmerzmittelverordnung beteiligt. An der Gesamtverordnung aller Arzneimittel eines Patienten waren im Mittel 3,3 Praxen beteiligt (Abb. 3).

Unabhängig von einer medikamentösen Behandlung suchten die Versicherten 7,9 Arztpraxen im ersten Jahr ihrer Opioidtherapie auf. Versicherte der Basispopulation der Erwachsenen ohne Krebsdiagnose (durchgängig versichert 2017 bis 2021) suchten im Jahr 2019 im Mittel 5,4 Praxen auf, also 2,5 Praxen weniger als Versicherte mit Langzeitopioidtherapie.

#### Multimorbidität bei Versicherten mit Langzeitopioidtherapie im ersten Therapiejahr

Bei Versicherten mit in 2019 neu begonnener Opioidtherapie und Langzeitopioidtherapie im ersten Therapiejahr (Studienpopulation 3a) wurden im Mittel im ersten Behandlungsjahr 13 verschiedene chronische Erkrankungen für einen Patienten dokumentiert. Die häufigsten Diagnosegruppen waren arterielle Hypertonie (ICD-10-Code: I10–I15) mit 68,6 % und Sonstige Krankheiten der Wirbelsäule und des Rückens (M50–M54) mit 53,3 % sowie Stoffwechselstörungen (E70–E90) mit 47,7 % (siehe Tabelle E4 im Online-Zusatzmaterial).

## Diskussion

75,8 % der BARMER-Versicherten mit einer Opioidverordnung im Jahr 2021 erhielten diese für die Behandlung von Nichttumorschmerzen. Die Prävalenzrate für eine mindestens einmalige Opioidverordnung bei Versicherten ohne Krebsdiagnose lag im Jahr 2021 bei den BARMER-Versicherten bei 5,7 %. In einer ebenfalls auf Routinedaten basierenden älteren Studie von Schubert et al. lag im Jahr 2010 der Anteil der Opioidempfänger ohne Hinweis auf eine Krebsdiagnose bei 76,7 % [28]. Ausgehend von der in dieser Studie ausgewiesenen Behandlungsprävalenz mit Opioiden von insgesamt 4,5 % kann hieraus eine Opioidbehandlungsprävalenz bei Nichttumorpatienten von 3,5 % ermittelt werden. In einer Studie von Häuser et al. (2018) zur Langzeitopioidtherapie bei Nichttumorschmerz auf Basis einer Stichprobe von 69 gesetzlichen Krankenkassen wurde für das Jahr 2014 eine Behandlungsprävalenz mit Opioiden von 4,2 % ermittelt [17]. Zum Vergleich: 17 % der US-Amerikaner erhielten mindestens eine Opioidverschreibung im Jahr 2017 [3].

Ein eindeutiger Anstieg der Langzeitverordnungen in Deutschland lässt sich nicht feststellen. In früheren Studien zeigte sich, dass 1,3 % der Versicherten der BARMER im Jahr 2012 [21] und 0,8 % der Versicherten von 69 gesetzlichen Krankenkassen im Jahr 2014 [17] eine Langzeittherapie mit Opioiden erhielten, definiert als mindestens je eine Verordnung in drei zusammenhängenden Quartalen. Die Kriterien einer Langzeitanwendung im BARMER Arzneimittelreport 2023 [11] waren weniger strikt, nämlich eine Behandlung mit mehr als 91 Tagesdosen („defined daily dose“ [DDD]) über einen Zeitraum von über 91 Tagen. 1,9 % der BARMER-Versicherten erfüllten 2021 diese Kriterien. 62,9 % dieser Personen hatten jährliche Opioidverschreibungen in den vergangenen fünf Jahren, sodass eine Langzeitanwendung über diesen Zeitraum bei 1,2 % der Versicherten angenommen werden kann.

Die Alters- und Geschlechtsanalysen der Opioidverschreibungen des BARMER Arzneimittelreports sind in Übereinstimmung mit den Ergebnissen aus repräsentativen Bevölkerungsstichproben der Jahre 2011 und 2013, nämlich dass chronische Schmerzen mit dem Alter zunehmen und Frauen häufiger wegen Schmerzen mit Medikamenten behandelt werden als Männer [12, 16]. Der beobachtete Rückgang im Anteil mit Langzeitopioidbehandlung über fünf Jahre bei den über 80-Jährigen könnte dadurch bedingt sein, dass die Therapie später begonnen hat und z. B. bei palliativem Einsatz nur eine kurze Zeit stattfand („end of life“).

Die meisten Opioide wurden im hausärztlichen Bereich verschrieben und auch die Therapie wurde dort initiiert. Der Anteil von Versicherten, die Verordnungen von Anästhesisten (Surrogatparameter für Schmerztherapeuten) ausgestellt bekamen, lag in den BARMER-Daten bei Versicherten mit Langzeitverordnungen in 2021 bei 10,7 %. In einer Studie basierend auf einer repräsentativen deutschen Bevölkerungsstichprobe im Jahr 2013 gaben 18 % der Personen mit chronischem nichtbeeinträchtigendem Nichttumorschmerz und 32 % der Personen mit beeinträchtigendem Nichttumorschmerz eine Mitbehandlung durch einen Schmerztherapeuten an [16].

Die am häufigsten verschriebenen Opioide in der BARMER-Stichprobe 2021 waren mit großem Abstand Tilidin und Tramadol, gefolgt mit gleicher Häufigkeit von Fentanyl und Oxycodon. Die häufigen Verschreibungen von Tilidin und Tramadol können durch die fehlende Betäubungsmittelrezeptpflichtigkeit (Ausnahme Tilidintropfen) und ihren Nimbus als sogenannte „schwache“ Opioide (mit der Assoziation „weniger nebenwirkungsreich“) erklärt werden.

Nur von einer Ärztin oder einem Arzt erhielten 36,6 % der Versicherten mit Langzeittherapie Verordnungen von opioid- und nichtopioidhaltigen Schmerzmitteln innerhalb eines Jahres nach Beginn der Opioidtherapie. Bei rund 60 % besteht somit ein erhöhtes Risiko, dass es zu nicht intendierten Doppelverordnungen oder risikoreicher Komedikation kommen kann, sofern kein vollständiger Medikationsplan vorliegt, der von den verschreibenden Ärzten eingesehen und bei Neuverschreibungen oder Dosismodifikationen aktualisiert wird.

Die – auch längerfristige – Komedikation mit Nichtopioiden, Antikonvulsiva und/oder Antidepressiva erfordert ein Monitoring (z. B. anticholinerge Belastung bei Komedikation mit Antidepressiva) sowie eine regelmäßige Aktualisierung des Medikationsplans der Patienten. In Leitlinien wird auf die Gefahr erhöhter Mortalität (Risiko für Atemdepression und Opioidüberdosierung) z. B. bei Komedikation mit Antikonvulsiva hingewiesen [14, 29].

Bei Patienten mit Multimorbidität und einer Indikation für Opioide besteht ein höheres Risiko für Wechsel- und Nebenwirkungen, die ein engmaschiges Monitoring erfordern. Zugleich weist der hohe Anteil von Opioidpatienten mit Multimorbidität darauf hin, dass Opioide bei vulnerablen Personen zum Einsatz kommen. In Anbetracht dieser Multimorbidität und daraus resultierender weiterer Polypharmazie vieler Patienten mit akuten und chronischen Schmerzen ist eine gemeinsame Entscheidungsfindung mit den Patientinnen und Patienten und gegebenenfalls ihren Angehörigen über ihre Präferenzen und Prioritäten bei der Behandlung erforderlich. Die in Aktualisierung befindliche Living Guideline der Deutschen Gesellschaft für Allgemeinmedizin (DEGAM; [27]) gibt Hilfestellung für die Gesprächsführung mit dem Patienten und zur Optimierung der Arzneimitteltherapie. Die Medikation für internistische und neurologische Erkrankungen zusammen mit den verschiedenen Schmerzmitteln kann bei einzelnen Patienten zu risikoreichen Arzneimittelinteraktionen führen. Hinweise auf vermeidbare Risiken der Opioidtherapie und Empfehlungen zur Risikominimierung gibt die S2k Living Guideline „Arzneimitteltherapie bei Multimorbidität“ [10] der medizinisch-wissenschaftlichen Fachgesellschaften, koordiniert durch die Deutsche Gesellschaft für Innere Medizin.

### Limitationen

Bei der Bewertung der Ergebnisse sind einige Limitationen zu beachten. So weist die Versichertenpopulation der BARMER einen höheren Anteil von Frauen und nicht berufstätig Versicherten auf als die Population aller gesetzlich Krankenversicherten. Da Frauen ein anderes Inanspruchnahmeverhalten als Männer aufweisen, ist bei nicht alters- und geschlechtsstratifizierten Auswertungen von höheren Prävalenzen auszugehen. Auch kann nicht ausgeschlossen werden, dass Versicherte anderer Kassen ein anderes Inanspruchnahmemuster zeigen [18].

Aus Routinedaten ist nicht ersichtlich, ob die verordnete Medikation auch eingenommen wurde. Immerhin deutet die Einlösung des Rezepts darauf hin, dass die Absicht bestand, das Medikament einzunehmen. Auch nicht eingelöste Rezepte können nicht erfasst werden. Insofern kann die Verordnung unter- und die Anwendung überschätzt werden. Die ärztlich verordnete Dosierung ist nicht in den Routinedaten angegeben. Bei der Definition der Langzeitanwendung über die Anzahl verordneter DDD können Personen, die mit niedrigen Dosierungen therapiert werden, unter den geforderten 91 DDD bleiben, obwohl sie über einen langen Zeitraum Opioide in niedriger Dosierung erhalten. Damit würde die Langzeitanwendung von Opioiden unterschätzt. Auch lässt sich aus den Verordnungsdaten nur bedingt die Angemessenheit beziehungsweise Leitlinienkonformität der Opioidverschreibungen bestimmen.

Bei der Komedikation mit trizyklischen Antidepressiva als Koanalgetikum ist aufgrund niedrigerer Dosierung im Vergleich zu der bei der Indikation Depression international festgelegten Tagesdosis (DDD) von einer Unterschätzung des Zeitraums einer überlappenden Therapie auszugehen. In der hier durchgeführten Analyse wurden bei der Verordnung der Antikonvulsiva und Antidepressiva nicht die Indikationen berücksichtigt, da der Fokus auf der Häufigkeit zusätzlicher Therapie und damit einhergehender möglicher Risiken lag.

Verschreibungen durch andere Ärzte können durch Urlaube oder Krankheit des primären ärztlichen Ansprechpartners des Patienten für die Opioidverschreibung bedingt sein und sind nicht immer ein Hinweis auf „doctor hopping“. Eine hohe Anzahl an Verschreibenden kann jedoch auch als Hinweis auf Therapieprobleme gewertet werden.

## Fazit für die Praxis

- Es gibt keinen Hinweis auf steigende Langzeitopioidverordnungen bei nichttumorbedingten Schmerzen in Deutschland in den letzten 10 Jahren.
- Opioide gegen nichttumorbedingte Schmerzen werden überwiegend von Hausärzten verschrieben. Schmerzmedizinische Fortbildungen im hausärztlichen Bereich, z. B. im Rahmen eines Kurses in schmerzmedizinischer Grundversorgung, sind sinnvoll.
- Die häufige Verordnung von Schmerzmitteln durch verschiedene Fachgruppen erfordert die kontinuierliche Aktualisierung des Medikationsplans durch alle Verschreibenden mit dem bundeseinheitlichen Medikationsplan.
- Vor der Verschreibung von Opioiden (und Nichtopioiden) sollten mit älteren und/oder multimorbiden Patientinnen und Patienten ihre Präferenzen besprochen und die Behandlungsprioritäten gemeinsam definiert werden.
- Bei der Verschreibung von Opioiden sind mögliche Arzneimittelinteraktionen, vor allem bei Menschen mit Multimorbidität und Polypharmazie, zu überprüfen.
- Die von der Deutschen Schmerzgesellschaft koordinierte S3-Leitlinie zur Langzeitanwendung von Opioiden bei chronischen nichttumorbedingten Schmerzen gibt Hilfestellung in Bezug auf die Indikationsstellung für Opioide, das Monitoring der Therapie, die Dosisanpassungen bei Leber- und Niereninsuffizienz, die Kombination mit anderen zentral wirksamen Medikamenten und die Einbindung in ein multimodales Therapiekonzept.

## Supplementary Information

Opioide: Wirkstoffe, Komedikation, Verordner, Diagnosen

## Data Availability

Die Daten sind aus Datenschutzgründen nicht frei zugänglich. Der Datenzugang setzt eine Antragsstellung bei der BARMER voraus.
