# Supplementary material for: Opioid prescriptions for insured individuals without cancer in Germany: data from the BARMER
Source: Schmerz. 2024 Dec 5;39(5):359–68. [Article in German] doi: 10.1007/s00482-024-00852-8 (PMC12446139; doi:10.1007/s00482-024-00852-8)
Supplement: Supplementary file 1 — Opioide: Wirkstoffe, Komedikation, Verordner, Diagnosen [file 482_2024_852_MOESM1_ESM.pdf]

Online-Zusatzmaterial

Opioidverordnungen bei Versicherten ohne Krebserkrankung in Deutschland – Daten der BARMER

Tabelle E1: Häufigste bei Erwachsenen verordnete Opioide im Fünfjahreszeitraum 2017 bis 2021

ATC-Kode	Wirkstoff*	Anteil Versicherte mit Langzeitverordnung in 2021 und Verordnung in vier Jahren davor in Prozent		
		Gesamt	Männer	Frauen
N02AX01	Tilidin	0,6	0,4	0,8
N02AX02	Tramadol	0,3	0,2	0,4
N02AA05	Oxycodon	0,3	0,2	0,4
N02AB03	Fentanyl	0,2	0,1	0,3
N02AA03	Hydromorphon	0,2	0,1	0,2
N02AX06	Tapentadol	0,2	0,1	0,2
N02AA01	Morphin	0,1	0,1	0,1
N02AE01	Buprenorphin	0,1	0,1	0,1

*Wirkstoffe, die mindestens ein Promille der Versicherten in 2021 verordnet bekamen. Wirkstoffe von Kombinations-ATC-Kodes werden einzeln bei den entsprechenden ATC-Kodes berücksichtigt. Quelle: BARMER-Daten 2017–2021; Studienpopulation 1a: BARMER-Versicherte 2021 ohne Krebsdiagnose ab 18 Jahre, durchgängig versichert 2017 bis 2021, gesamt n = 6.049.276, Männer n = 2.526.955, Frauen n = 3.522.321

Tabelle E2: Langzeitkomedikation über mindestens 91 Tage mit Antikonvulsiva, Antidepressiva beziehungsweise Nicht-Opioid-Analgetika bei Versicherten mit Langzeitopioidtherapie in 2021

Geschlecht	Altersgruppe	Anteil Versicherte mit Langzeitopioidtherapie in 2021 und Komedikation* mit… dem Wirkstoff/der Wirkstoffgruppe (weitere Wirkstoff(gruppen) möglich): Pregabalin/ Gabapentin	Anti-depres-siva	NSAR	Meta-mizol	NSAR/ Metamizol	nur den aufgeführten Wirkstoffgruppen in 2021 zu verschiedenen Zeiten oder gleichzeitig: Preg./Gabp. Antidepress.	Preg./Gabp. NSAR/Metamizol	Antidepressiva NSAR/Metamizol	Preg./Gabp. Antidepressiva NSAR/Metamizol
Männer	18-64	15,8	27,8	19,1	7,6	25,2	4,7	2,7	5,5	2,8
	65-79	14,7	20,7	13,0	9,6	21,6	3,3	2,7	4,1	1,6
	80+	9,8	14,8	6,7	13,4	19,1	1,4	2,2	3,2	0,8
	Gesamt	14,0	22,2	14,0	9,7	22,5	3,4	2,5	4,4	1,9
Frauen	18-64	16,3	37,2	22,6	8,0	28,6	5,4	2,6	8,7	3,5
	65-79	11,6	26,2	14,3	9,7	22,7	3,0	2,0	5,5	1,8
	80+	6,6	20,1	6,7	15,3	20,9	1,1	1,5	5,5	0,8
	Gesamt	10,4	26,0	12,7	11,9	23,3	2,7	1,9	6,3	1,7
Ges.	Gesamt	11,4	24,9	13,0	11,3	23,0	2,9	2,1	5,8	1,8

*Parallele Verordnung über mindestens 91 Tage. Quelle: BARMER-Daten 2020–2021; Studienpopulation 2: BARMER-Versicherte 2021 ohne Krebsdiagnose ab 18 Jahre mit Langzeitopioidtherapie in 2021, gesamt n = 126.188, Männer n = 34.834, Frauen n = 91.354

Tabelle E3: Fachgebiete der die inzidente Opioidverordnung in 2019 ausstellenden Ärztinnen und Ärzte

Fachgebiet	Anteil Versicherter mit Erstverordnung Opioid ausgestellt von Arzt/Ärztin des Fachgebiets bei Versicherten mit Therapiebeginn in 2019 – Alle Altersgruppen – Gesamt Prozent	Männer Prozent	Frauen Prozent	Beide Geschlechter nach Altersgruppen in Jahren – 18-64 Prozent	65-79 Prozent	80+ Prozent
Hausärztin / Hausarzt	59,5	58,1	60,3	54,5	62,3	73,8
Orthopädie	17,0	16,4	17,3	17,6	18,2	12,6
Chirurgie	7,2	7,9	6,8	8,4	6,5	4,1
Zahnheilkunde	6,3	7,7	5,6	8,7	3,8	1,6
unbekannt	3,6	4,1	3,4	4,2	3,0	2,7
Anästhesiologie	1,3	1,0	1,4	1,3	1,3	1,0
Neurologie	0,8	0,7	0,9	0,8	1,0	0,7
Physikal./Rehabilitative Medizin	0,8	0,7	0,9	0,8	0,9	0,5
Innere Medizin	0,6	0,5	0,6	0,4	0,7	1,2
Hals-Nasen-Ohrenheilkunde	0,4	0,4	0,4	0,6	0,1	0,1
Sonstige Fachgebiete	2,5	2,6	2,5	2,8	2,3	2,0

Quelle: BARMER-Daten 2017–2021; Studienpopulation 3: BARMER-Versicherte ohne Krebsdiagnose ab 18 Jahre mit neu angesetzter Opioidtherapie in 2019, durchgängig versichert 2017 bis 2021, gesamt n = 142.598, Männer n = 49.759, Frauen n = 92.839

Tabelle E4: Chronische Erkrankungen (Diagnosegruppen) bei inzidenten Opioidpatienten in 2019 mit Langzeitopioidtherapie in einem Jahr ab Inzidenz

ICD-10-Gruppe*	Diagnosegruppe	Anteil Versicherter mit der jeweiligen Diagnose in Prozent		
		Gesamt	Männer	Frauen
I10-I15	Hypertonie [Hochdruckkrankheit]	68,6	64,8	70,2
M50-M54	Sonstige Krankheiten der Wirbelsäule und des Rückens	53,1	54,4	52,5
E70-E90	Stoffwechselstörungen	47,7	46,4	48,2
R50-R69	Allgemeinsymptome	47,1	38,5	50,7
M15-M19	Arthrose	44,0	35,4	47,7
Z80-Z99	Personen mit potentiellen Gesundheitsrisiken aufgrund der Familien- oder Eigenanamnese und bestimmte Zustände, die den Gesundheitszustand beeinflussen	42,3	40,2	43,1
M45-M49	Spondylopathien	36,8	35,9	37,2
I30-I52	Sonstige Formen der Herzkrankheit	31,5	29,9	32,2
F40-F48	Neurotische, Belastungs- und somatoforme Störungen	29,6	25,7	31,3
F30-F39	Affektive Störungen	29,2	22,8	31,9
E10-E14	Diabetes mellitus	29,2	33,3	27,4
E00-E07	Krankheiten der Schilddrüse	28,9	14,0	35,1
I80-I89	Krankheiten der Venen, der Lymphgefäße und der Lymphknoten, anderenorts nicht klassifiziert	25,3	16,3	29,1
K20-K31	Krankheiten des Ösophagus, des Magens und des Duodenums	24,4	23,2	24,9
R25-R29	Symptome, die das Nervensystem und das Muskel-Skelett-System betreffen	23,6	16,1	26,7
G40-G47	Episodische und paroxysmale Krankheiten des Nervensystems	22,8	22,2	23,1
M40-M43	Deformitäten der Wirbelsäule und des Rückens	22,2	18,2	23,9
J40-J47	Chronische Krankheiten der unteren Atemwege	22,0	21,8	22,0
M70-M79	Sonstige Krankheiten des Weichteilgewebes	21,9	19,6	23,0
E65-E68	Adipositas und sonstige Überernährung	20,8	22,1	20,2
M80-M85	Veränderungen der Knochendichte und -struktur	20,1	6,3	26,0

*Diagnosegruppen, die bei mindestens 20 Prozent der BARMER-Versicherten mit inzidenter Opioidverordnung 2019 und Langzeitopioidtherapie im ersten Jahr ab Inzidenz ambulant (gesicherte Diagnosen) und stationär (Haupt- und Nebendiagnosen) dokumentiert waren. Chronische Erkrankung: Diagnosegruppe musste beim Versicherten in mindestens drei von vier Quartalen dokumentiert sein. Quelle: BARMER-Daten 2020–2021 (Grandt et al. 2023; Tab 2.23 [1]); Studienpopulation 3a: BARMER-Versicherte 2021 ohne Krebsdiagnose ab 18 Jahre mit Langzeitopioidtherapie in einem Jahr ab Inzidenz in 2019, durchgängig versichert 2017 bis 2021, gesamt n = 12.690, Frauen n = 8.922, Männer n = 3.768

Literatur

1. Grandt D, Lappe V, Schubert I (2023) Arzneimittelreport 2023: Medikamentöse Schmerztherapie nicht-onkologischer ambulanter Patientinnen und Patienten BARMER, Berlin
